# Round the Hospitals

**Published:** 1921-07-09

**Authors:** 


### ROUND THE HOSPITALS.

Judging by the large number of visitors to the Bazaar organised by the London Hospital nurses on Wednesday last, the nucleus of the fund towards reopening the closed beds should prove a substantial one. Even before the formal opening at noon by Queen Alexandra—who drove down the Whitechapel Road amid the cheers of the neighbourhood—many visitors were buying goods at the picturesque stalls from the dainty servers in their print frocks and starched caps. There was something to please even the most fastidious: fancy work, china and glass, baskets and trays, antiques, pictures, ices, and fruit. Each stall and sideshow bore its own painted signpost, the skilful work of a member of the nursing staff. In the Village Fair, in charge of the medical staff, the young visitors revelled in donkey-rides, houp-la, Punch-and-Judy show, cocoanut-shies, while their elders

**ROUND THE HOSPITALS**—*(continued)*.

went on "personally conducted tours" round the hospital wards, out-patient department, and nurses' home. Old and young alike lingered round the livestock—a couple of goats tethered with red and blue ribbons, while fluffy kittens were on show. It was reminiscent of war-time to see the queue lined up to view the dolls-house sent by Queen Mary and the model of the Cenotaph presented by the Prince of Wales. We feel sure that from Her Majesty down to the patients, eagerly watching the fun from the balconies, everyone enjoyed the London Hospital Fête.

The retirement of Miss Colebrooke, affectionately known as "Granny," from her post as Sister Casualty at St. Mary's Hospital, Paddington, has occasioned great tribulation among her colleagues accustomed to rely on her unfailing resourcefulness, urbanity, and tranquillity of spirit. She has been thirty-four years in the service of the hospital, and it is inspiriting to be told, by common consent of the residents, who alone come into contact with her work, that after all these years she is "as efficient as ever." Her name will long live in the annals of the hospital, and we have no doubt of her proving as cheering an influence to a smaller circle in her retirement as she has been during the crowded and engrossing years of her valuable hospital career.

The courageous and hopeful note sounded in the thirty-first annual report of Queen Victoria's Jubilee Institute for Nurses is worthy of all praise, and should serve as an example to the many societies now labouring under after-war difficulties. The work has grown beyond all precedent. The number of nurses placed on the Roll during the year was 378, the largest number since the founding of the Institute, and seventy-five former Queen's Nurses rejoined, making the number of Queen's Nurses at work on January 1, 1921, in Great Britain and Ireland 1,936. Including village nurses and midwives over 5,000 were at work under the Institute. The new rates of salary are producing their effect in inducing nurses to enter the service, and in a short time it is hoped to meet all the demands received for Queen's Nurses. The inspectors and superintendents constitute a valuable body of nursing sisters in touch with all the numerous developments of the National Health Service, and the opportunity for obtaining posts of this description should act as an inducement to women of advanced education to undertake this branch of work. It is necessary to raise an additional £12,000 a year if the Institute is to be kept working as at present. We suggest that some of the burden should be shared by the Nursing Associations, which for a trifling affiliation fee benefit by the training centres. There are over a thousand of these Associations, and £5 from each would go some way to solve the financial difficulty. Some have already sent contributions.

The training courses organised by the National Health Society to prepare health visitors and infant-welfare workers for their duties commence in September, and particulars have already reached us which our readers will do well to study. Students with no previous knowledge or experience must spend two years over the course, which is crowned by an examination and certificate, and pay a fee of £50. Hospital nurses, certificated after three years' training, students with a University degree, and health visitors with at least three years' full-time experience may obtain their certificate after a shortened course of one year, the fee for which is £25. The courses conform to the Regulations of the Board of Education and Ministry of Health, and provide a recognised passport to public-health appointments of a lucrative nature. The central situation, at 53 Berners Street, W. 1, of the lecture-room of the National Health Society renders these courses very convenient and accessible.

The hospitals are not remaining idle in regard to the College of Nursing recommended scale of salaries. Sir Arthur Stanley, in his speech at the annual meeting of the College at Edinburgh, indicated the desire of hospitals to adopt this standard, and we understand that a meeting has been arranged between the Salaries Committee of the College and a deputation from the British Hospitals Association to discuss the matter. The British Hospitals Association is also taking steps in regard to the Draft Syllabus of the General Nursing Council, and at its annual meeting on July 1 (reported elsewhere in this issue) it passed a resolution that the General Nursing Council should be requested not to put their Syllabus definitely in operation until the opinion of the hospitals has been heard.

Great is the dismay of the Hackney Guardians at the Syllabus issued by the General Nursing Council. It is recognised very clearly by the Infirmary Committee that the appointment of a sister-tutor is necessary if the course of training laid down in the Syllabus is to be carried out. In the present plight of the ratepayers the proposal to appoint a new salaried officer has naturally met with considerable opposition, and the Infirmary Committee have their report referred back. Nevertheless, we hope they will be able to show the Board that the proper training of their probationers under a sister selected for her teaching powers will make in the end for economy.

The Serbian Ministry of Health is co-operating with the League of Red Cross Societies at Geneva to defray the cost of engaging Miss Enid Newton, R.R.C., a former Guy's nurse, to direct the nursing services in Serbia, establish centres for maternity and child-welfare work, and develop the training of Serbian women as nurses. In almost untried enterprises of this description, gifts of heart and of head are almost equally needed, a tireless patience, and, above all, unquenchable faith in the possibilities of human nature. Miss Newton's previous experience as director of nursing

**ROUND THE HOSPITALS**—(*continued*).

at home and abroad, in peace and in war, will stand her in good stead. We wish her all success, and do not doubt her attaining it.

The nursing staff at the Holborn and Finsbury Hospital, Highgate, have petitioned the Guardians not to discontinue the payment of war bonus as decided at a recent meeting. The petition has not been received with favour, and it would seem that isolated action in this matter is little good. The nurses' claim for the continuance of the war bonus, or rather for its addition to salary, stands on quite a different basis from that of other workers. Admittedly before the war their pay was wretchedly inadequate. Even with the war bonus nursing salaries are lower than those in any other branch of work requiring long training and certification. Great dislocation of the nursing services, and a still further and much more acute shortage of probationers, will result if salaries undergo a general decrease in September by the cessation of war bonus. It is a matter which calls urgently for outside intervention, and we hope to hear that the College of Nursing has it under consideration. The number of their Poor-law trained members is considerable.

The Draft Rules of the General Nursing Council for Scotland, of which we gave an abstract in our last issue, provide, as we there specified, no fewer than five alternative standards of training for "existing nurses," and it must be generally recognised that the danger of flooding the Register with unskilled women has been completely avoided. No one at all approximating to the unfortunate "qualified nurse" of the Irish rules can be admitted, and the far-sighted policy of the Scottish Local Government Board in providing for the regular training, examination, and certification of the members of the service is now bearing fruit. At the same time the interests of all regular nurses are amply safeguarded. It should be generally known, though we fear it is often forgotten, that there are at present few, if any, nurses in reputable *bona-fide* practice who have not received some kind of institutional training for their duties extending over a year or two.

Miss Kate E. Ray, Matron of St. Mary's Hospital for Women and Children, Plaistow, has opened a special fund for the Nurses' Home, so urgently required, at that institution. At present wards which can ill be spared are being used as dining-rooms, and the comfort of the nurses is greatly interfered with, owing to the deficient accommodation. Princess Marie Louise has taken an active part in supporting the appeal, which is impeded by the other requirements of the hospital. To build the new Out-patients' Department and the Nurses' Home a sum of £70,000 is needed.

Matrons and sisters will do well to remember that they can procure all the books and other literature they desire to provide for the patients under their care from the Red Cross War Library, which, at 48 Queen's Garden, W. 2, is continuing on behalf of civilian hospitals the good work carried on during the war by Mrs. Gaskell for the hospitals in the fighting area. The library aims at supplying all hospitals, both London and provincial, with as many books and as often as is required. This can only be done if every owner and buyer of books will remember to send such as they have no further use for, or can spare, to the headquarters at Queen's Gardens. A postcard will always bring a sack, so that at the least trouble to the sender the books can be transferred from the bookshelves to the patient's bed, via the clearing-house at Queen's Gardens.

The need for a new wing at the Sidcup Cottage Hospital has resulted in a very interesting experiment. It is being built by twenty Sidcup volunteers, who have signed on to give their spare time, under a competent foreman, to the bricklaying and other labour involved. They include the local bank manager, the President of the hospital, Mr. Rosevear, one of the churchwardens, an engineer, and several city men. Some of the nurses have volunteered to help, and a photograph in the daily press shows them busy mixing mortar.

We note with satisfaction that at the Devonshire Hospital and Buxton Bath Charity, the minimum scale of salaries, as recommended by the Salaries' Committee of the College of Nursing, has been recently adopted. Volunteer nurses have generously placed their services at the disposal of the matron, Miss M. Gilkes-Robinson, R.R.C., and have given much-appreciated assistance.

Miss E. Stewart, who is retiring at the end of July from her post as matron of the Bow Infirmary, has had seven years added on to her superannuation allowance, which brings it to £185 per annum, subject to the consent of the Ministry of Health. Miss Stewart received a grant of £200 from the War Office as a slight recognition of her most valuable services at Homerton during the war. The work she has accomplished in her own institution is beyond all praise, and her place will be hard to fill.

Much disappointment has been occasioned to the nursing and massage staffs of Beckett's Park Hospital in Leeds under the Pensions Ministry by the withdrawal of the privilege, accorded in war time and since, of using one of the tennis courts and the swimming-bath attached to the training college in Beckett's Park. It seems that now all the students are again in residence at the training college they need all the recreation facilities which the estate affords. But surely there are periods in the day when the students are all engaged and members of the nursing staff who are off-duty might get a chance. It is a matter for friendly co-ordination of interests instead of hard-and-fast rules.

